# Polyaniline-Based Cationic Porous Organic Polymers for Fast and Efficient Anion-Exchange-Driven Capture of Cr_2_O_7_^2–^

**DOI:** 10.1021/acsapm.4c00658

**Published:** 2024-05-24

**Authors:** Long Pan, Zilu Liu, Marcos Villeda Hernandez, Bob C. Schroeder, Yuchen Sun, Charl F. J. Faul

**Affiliations:** † School of Chemistry, University of Bristol, Bristol, England BS8 1TS, U.K.; ‡ Institute for Advanced Pharmaceutical Materials, Asymchem Life Sciences (Tianjin) Co., Ltd., No.265 South Avenue, TEDA, Tianjin 300462, P. R. China; § Department of Chemistry, University College London, London WC1H 0AJ, U.K.

**Keywords:** water treatment, Cr(VI) removal, hydrophilic polymer, cationic porous organic polymer, poly(triphenylamine), conjugated microporous polymer

## Abstract

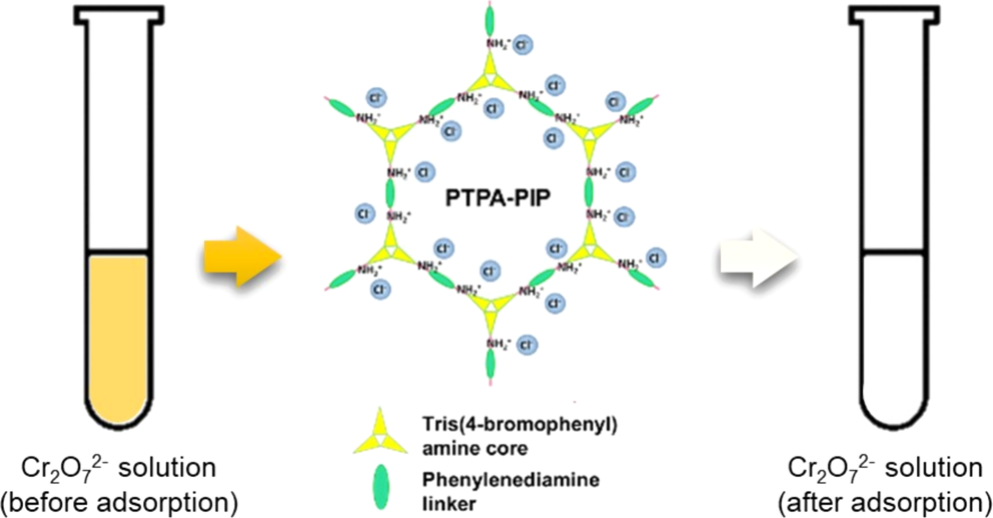

Efficient treatment of wastewater contaminated with carcinogenic Cr(VI) has been a long-term challenge for both academic and industrial research efforts. Removal of Cr(VI) species by ion exchange is a relatively simple and efficient method, and its combination with highly tailorable nanomaterials is promising for the treatment of such wastewater. Here, we report a type of cationic porous organic polymer (POP), namely, PTPA–PIP, which can be prepared simply by converting the corresponding aromatic polyamine PTPA to its protonated form, thereby significantly increasing its hydrophilicity and ability to disperse homogeneously in water, crucial for application in water treatment. In addition to detailed characterization of the physicochemical properties of PTPA–PIP (including using Fourier transform infrared (FTIR), X-ray photoelectron spectroscopy (XPS), Brunauer–Emmett–Teller (BET), and solid-state NMR techniques), adsorption experiments demonstrate that PTPA–PIP removes low-concentration dichromate anions with very high performance, including excellent exchange capacity (maximum capacity of 230 mg Cr_2_O_7_^2–^/g PTPA–PIP), ultrafast removal (initial adsorption rate of 83 mg g^–1^ min^–1^), excellent selectivity (∼10% loss of adsorption capacity in the presence of 40-fold concentration of competing anions), as well as superior reusability (reusable for at least 5 cycles without compromised performance). These results demonstrate that PTPA–PIP is an outstanding candidate for application in industrial settings for the effective removal of harmful Cr(VI) pollutants in wastewater.

## 1 Introduction

Water pollution is a serious environmental issue and of global concern, as also highlighted by the United Nations as one of their sustainable development goals (SDGs), SDG 6.^1^ Toxic pollutants, especially heavy metal ions such as Hg^2+^, Cd^2+^, As(III), and Cr(VI) species, are commonly present in industrial and agricultural sites.^2,3^ The discharge of wastewaters containing such toxins not only disrupts natural aquatic ecosystems, leading to bioaccumulation,^4^ but also represents significant health risks to humans through the contamination of potable water sources.^5^

Cr(VI) anions are of particular concern as they are carcinogenic and mutagenic to living systems^6,7^ and are well known to cause kidney damage,^8^ allergies,^9^ and nerve damage.^10^ The US Environmental Protection Agency (EPA) has included them in their priority pollutant list.^11^ Cr(VI) compounds are still widely used in various industries including leather tanning, electroplating, and textile dying,^12^ where tanning industries alone discharge ∼30–35 L of Cr(VI)-contaminated water for each kilogram of tanned leather produced.^13^ Therefore, development of effective strategies to remove Cr(VI) from wastewater streams is highly desirable and urgently needed.

To date, numerous techniques such as adsorption, liquid–liquid extraction, ion exchange, and electrodialysis have been employed for the removal of Cr(VI).^14−20^ Ion exchange has been considered a preferred technique owing to its low cost, high efficiency, as well as being comparatively simple and safe to deploy.^21,22^ In recent years, porous cationic framework-based materials, especially cationic metal–organic frameworks (MOFs), have been successfully developed to detect and remove Cr(VI) anions from wastewater.^23−26^ For example, Liu et al. developed an MOF material by reacting ZrCl_4_ with 2,3,5,6-tetrakis(4-carboxyphenyl)pyrazine (H4TCPP).^26^ Its initial adsorption rate of Cr_2_O_7_^2–^ is as high as 38.0 mg g^–1^ min^–1^, and it demonstrates a capture capacity of 149 mg g^–1^. No change in its crystal structure was observed after 48 h exposure to aqueous solution at pH 0 or 11, although long-term stability was not determined. Li et al. synthesized an MOF material using AgClO_4_ and 4,4′-bis(1,2,4-triazole).^23^ The initial adsorption rate of this MOF is not reported, but within the first hour of the adsorption experiment, the average adsorption rate is 108.0 mg g^–1^ h^–1^ (1.8 mg g^–1^ min^–1^), and the capture capacity is calculated to be 158 mg g^–1^. The material was reported to be stable in aqueous solution in a pH range from 0 to 10 for up to 24 h. Apart from such MOF-based materials, several other types of materials, such as PEI-modified natural polymeric materials, or polystyrene-based ion exchange materials, have also been successfully used for Cr(VI) anion removal.^27−30^

However, many of these materials lack physicochemical stability and tend to decompose during water treatment. For example, Zhou et al. stated that most zirconium-based MOF materials are vulnerable to alkali attack (although some improvements have been made to remediate this problem).^20^ In addition, the often difficult synthesis has hindered their large-scale fabrication and deployment.

In recent years, three-dimensional (3-D) nanomaterials based on covalently bonded building blocks rather than coordination bonds are emerging as a new research focus. These materials are classified as porous organic polymers (POPs), into which a wide range of functional groups can be incorporated, including charged cationic or anionic groups.^31−34^ The physicochemical properties, functional groups, and active sites of cationic POPs can be easily tuned by postsynthesis counterion exchange, which provides a promising route to wide application of these materials.^35,36^ Moreover, compared with cationic MOFs, cationic POPs are constructed from stronger, stable covalent bonds with high physicochemical stability in a variety of harsh operating environments,^37,38^ which provides a variety of options for further modification and application: These materials can be subjected to further chemical reactions in so-called postsynthesis modification approaches to add further functionality or expand their potential application areas.^39−41^ Consequently, these stable materials are potentially useful in a wide range of settings, including for the removal of metal anions, including Cr(VI) anions, from wastewaters.^42−44^

Recently, a few cationic POPs have been reported, but their applications have mainly focused on catalysis or gas adsorption/separation.^45−48^ However, a recent report by Wang et al. on Pb^2+^ adsorption in wastewater treatment featured a porous polyaniline framework that effectively removes Pb^2+^ to the ppb level.^49^ There are two main methods for the synthesis of cationic POPs: direct self-polymerization of ionic building blocks or copolymerization of ionic and neutral building blocks, as shown in Table S1.^50^ Several typical reactions for the synthesis of cationic POPs, such as Schiff-base formation,^51^ trimerization of −CN groups,^52^ Sonogashira–Hagihara reaction,^53^ and Friedel–Crafts alkylation reaction,^54^ have been used. However, the synthesis of ionic building blocks for self-polymerization or copolymerization is more complex than that for neutral moieties. Moreover, the conditions for such polymerization reactions, including solvents and catalysts, should be carefully chosen to match the polarity of the ionic building blocks, while preventing exchange reactions between the ionic building blocks and other ions from the reaction system.

In this work, we explore the design and development of a simple and convenient strategy for the synthesis of a cationic POP for the capture of Cr_2_O_7_^2–^ from wastewater. The material is based on a poly(triphenylamine) (PTPA) backbone, which is structurally similar to linear polyaniline. However, the trifunctional triphenylamine unit incorporated endows the resultant material with a 3-dimensional structure and self-supporting properties, and significantly higher surface area when compared with linear polyaniline. By facile postsynthesis treatment of the PTPA, the material can be converted to its cationic and hydrophilic derivative, namely PTPA–PIP. Once prepared, this material was investigated for its anion-exchange properties, specifically for the exchange and removal of Cr_2_O_7_^2–^ from contaminated wastewater, presenting a simple, facile, and promising materials solution to significant global challenges.

## 2 Experimental Section

All chemicals were obtained from Sigma-Aldrich (U.K.). PTPA was synthesized and optimized using the Bristol–Xi’an Jiaotong (BXJ) method,^55^ developed by our group, with a stoichiometric ratio of tris(4-bromophenyl) amine to phenylenediamine (1:1.5).^24^ Fourier transform infrared (FTIR) spectra were recorded on a PerkinElmer Spectrum 100 spectrometer. Thermogravimetric analysis (TGA) was carried out on a TGA Q500 apparatus under a nitrogen atmosphere (flow rate of 30 mL min^–1^) by heating (10 °C min^–1^) the samples to 800 °C. Scanning electron microscopy (SEM) was performed using a JEOL JSM-IT300 scanning electron microscope. X-ray photoelectron spectra (XPS) were obtained on a Thermo Scientific K-Alpha X-ray photoelectron spectrometer under ultrahigh vacuum (<5 × 10^–8^ Torr) and by using a monochromatic Al Kα X-ray source.

PTPA–PIP was prepared by immersion of PTPA (200 mg) in concentrated hydrochloric acid (20 mL) and stirring at room temperature for 12 h. The obtained suspension was centrifuged, and the residue was washed with 20 × 3 mL of water. The expected PTPA–PIP was obtained by drying overnight in vacuo at 50 °C.

A standard Cr_2_O_7_^2–^ solution (chromate (as Cr(VI)) 1000 mg/L in water) was purchased from VWR International Ltd. The solutions with Cr_2_O_7_^2–^ concentrations of 10, 15, 20, and 50 ppm of Cr(VI) were prepared by diluting the aforementioned standard solution. Ultraviolet–visible (UV–vis) absorbances at 257 and 350 nm of each prepared solution were recorded and used to construct the calibration curve. Fitting parameters were then used to predict the remaining Cr_2_O_7_^2–^ in the solution after adsorption.

All Cr_2_O_7_^2–^ adsorption measurements were conducted in the aqueous phase under ambient conditions. For measurement of maximum adsorption capacity, 15 mg of PTPA–PIP was suspended in 8 mL of Cr_2_O_7_^2–^ solution (500 ppm) at room temperature (with the unmodified PTPA used as a comparison). UV–vis measurements were used to monitor the concentration change in the supernatant, using the characteristic absorption of Cr_2_O_7_^2–^ at 257 nm.^56^ The adsorption isotherm was measured by adding 8 mg of PTPA–PIP to 8 mL of Cr_2_O_7_^2–^ solutions with different initial Cr(VI) concentrations (10, 25, 50, 75, 100, 150, and 200 ppm, respectively). After shaking for 12 h, the supernatants were extracted, and the equilibrium Cr_2_O_7_^2–^ concentrations were determined by UV–vis spectroscopy. For selective adsorption investigations, PTPA–PIP (10 mg) was immersed in an aqueous solution of K_2_Cr_2_O_7_ (100 ppm, 10 mL) in the presence of competing ions (Cl^–^, SO_4_^2–^, and NO_3_^–^) with *n*-fold (*n* = 1, 2, 4, 8, 10, 20, 40) concentrations. After 30 min, 1 mL of the solution was pipetted and used to determine the Cr(VI) concentration by UV–vis spectroscopy.

## 3 Results and Discussion

**Figure 1:**
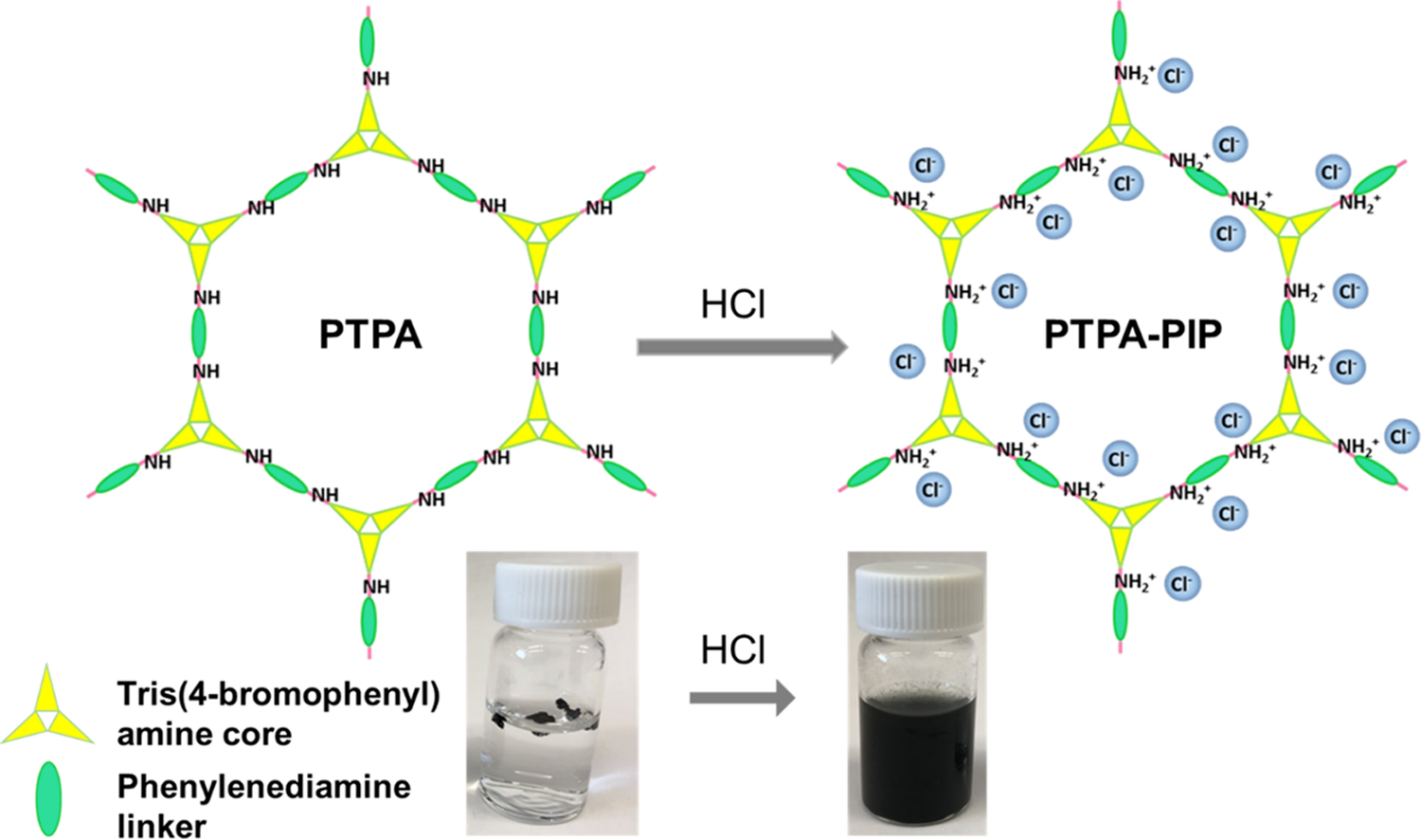
Schematic representation of the structure of PTPA–PIP, with images below showing the dramatic postdoping increase in dispersibility.

The synthesis of PTPA using the BXJ approach has already been reported by our group.^55^ As shown in Figure 1, after a simple and convenient postsynthesis treatment, PTPA–PIP, a doped poly(aniline)-like cationic POP, can be easily obtained through reaction between the amines and hydrochloric acid, analogous to the well-known doping of linear poly(aniline). This postsynthesis treatment method provides a simple strategy for the preparation of cationic POPs and offers cost-effective advantages such as ambient preparation conditions, no catalyst, and high yield, all essential factors for scale-up and potential industrial use of such cationic POPs. The hydrophobic nature of most POPs hindered their usability in water treatment to date as they do not disperse well in water. As shown in Figure 1, the postsynthesis treatment approach switches the polymer from hydrophobic to hydrophilic, enabling dispersibility and thus opportunities for wastewater treatment. SEM images of the polymers are presented in Figure S1, showing micrometer-sized amorphous polymer particles for both samples.

**Figure 2:**
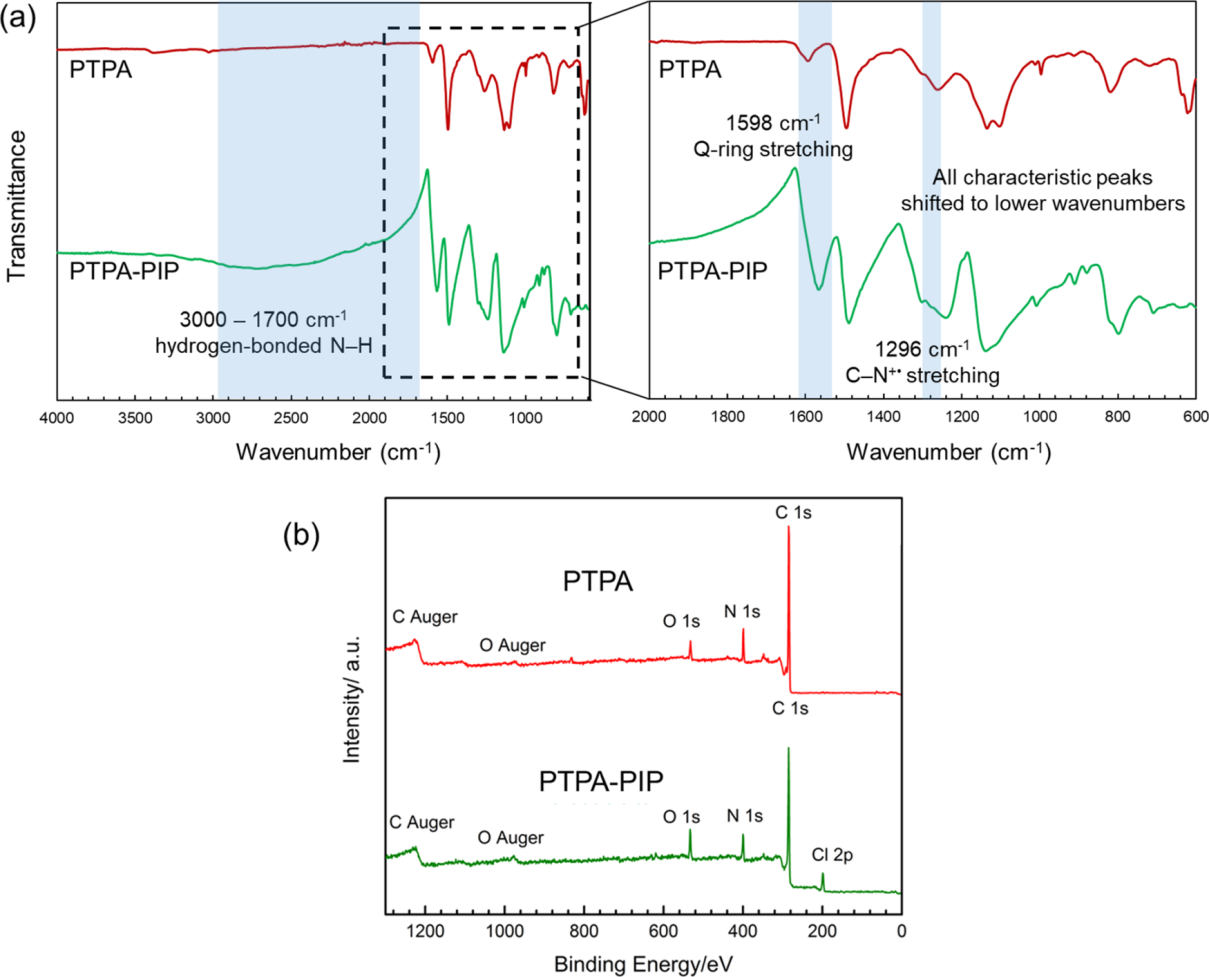
(a) FTIR spectra of PTPA and PTPA–PIP (see text for peak assignments). (b) X-ray photoelectron spectra of PTPA and PTPA–PIP. All spectra have been offset by an arbitrary constant.

The FTIR spectra of PTPA and PTPA–PIP are shown in Figure 2. For PTPA, the characteristic peaks observed at 1598 and 1497 cm^–1^ are attributed to quinonoid (Q) ring stretching and benzenoid (B) ring stretching, respectively. The band characteristic of C–N stretching in a BBB unit (the tertiary amine in the middle of three benzene rings) is observed at 1265 cm^–1^. A strong and broad band centered at 1134 cm^–1^ is assigned to the signal of the aromatic C–H in-plane deformation. The spectrum of PTPA–PIP exhibited a broad peak between 3000 and 1700 cm^–1^, which is attributed to the hydrogen-bonded N–H stretching vibration of −NH_2_^+^–. A shoulder peak at 1296 cm^–1^ is ascribable to a C–N^+•^ stretching vibration in the polaron structure. Similar characteristic peaks as described for PTPA were also detected for PTPA–PIP. Compared with PTPA, owing to the decreased electron cloud density, reduced force constants between atoms, and better conjugated structure of PTPA–PIP, all of the peaks shifted to lower wavenumbers (the peaks at 1598, 1497, 1260, and 820 cm^–1^ shifted to 1568, 1487, 1240, and 802 cm^–1^, respectively). In addition, the intensity ratio of the quinonoid (Q) ring stretching and benzenoid (B) ring stretching peaks can be used to evaluate the degree of doping of the polymers.^57^ For PTPA–PIP, the Q-ring stretching signal (1568 cm^–1^) is significantly stronger than that for PTPA (1598 cm^–1^), thus proving that PTPA–PIP was doped successfully and the product is consistent with the proposed structures.

Data obtained from X-ray photoelectron spectroscopy (XPS), as shown in Figure 2, were used to validate the elemental composition of the polymers and the elemental contents of PTPA and PTPA–PIP (Table 1). The new emerging peak at 199 eV, which was assigned to Cl 2p, is direct and unambiguous evidence for the success of our doping approach. The conversion ratio from amine to ammonium is 89%, which can be easily calculated by using the data of elemental content and the following formula:
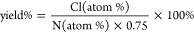
1In the solid-state ^13^C NMR spectra of PTPA and PTPA–PIP (Figure S2), two main resonances at 141 and 127 ppm were observed for both spectra and are assigned to the substituted and unsubstituted aromatic carbons, respectively. Compared with PTPA, the shapes of the peaks change from two individual sharp peaks to a broad peak with a shoulder peak because of the decreased electron cloud density.

**Table 1:** Elemental Content and Peak Position of Constituent Atoms in PTPA and PTPA–PIP

	PTPA				PTPA–PIP			
element	C	N	Cl	O	C	N	Cl	O
peak position (eV)	285	400	199	533	285	400	199	533
atom%	85.23	10.37	0	4.39	79.3	8.4	5.6	6.4

To further confirm the change in charge properties after treatment, ζ-potential measurements were performed for PTPA and PTPA–PIP. According to the results (see Figure S3), PTPA–PIP exhibits a positive charge in the aqueous environment, specifically +4.08 mV; while, in contrast, the untreated PTPA exhibited a negative charge of −16.1 mV. These results highlight a significant shift in surface charge, originating from the change in the chemical structure associated with the ionization of N atoms by acid treatment in PTPA–PIP.

The porosities and surface areas of PTPA and PTPA–PIP were investigated by nitrogen adsorption experiments at 77 K (Figure S4). Type-III nitrogen sorption isotherms, according to the IUPAC classification, were observed for the two polymers. The BET specific surface area (SSA) values are 80 and 25 m^2^ g^–1^, respectively. This decrease in SSA can be explained by the density increase of the cationic POP and the existence of abundant Cl^–^ anions blocking the pores.

**Figure 3:**
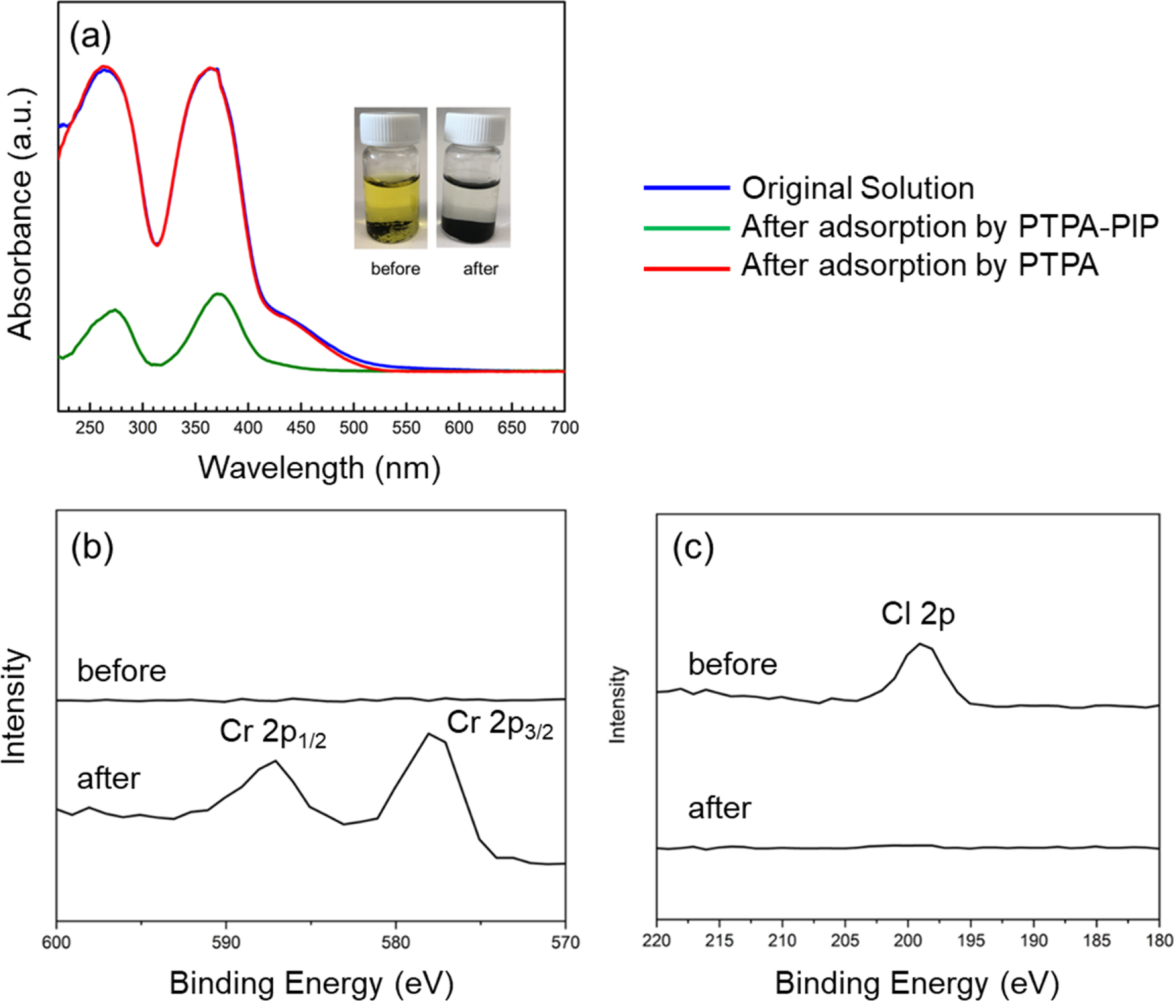
(a) UV–vis spectra of 500 ppm of aqueous Cr_2_O_7_^2–^ solutions before (blue) and after adsorption by PTPA (red) and PTPA–PIP (green). Inset: color change of the Cr_2_O_7_^2–^ solution before and 12h after exposure to PTPA–PIP. (b, c) High-resolution X-ray photoelectron spectra of PTPA–PIP before and after adsorption of Cr_2_O_7_^2–^: Cr 2p_1/2_ and Cr 2p_3/2_ (b) and Cl 2p (c).

As a polyaryl cationic polymer without other labile functional groups, PTPA–PIP should possess outstanding chemical and physical stability, prompting exploration for use as an adsorbent and investigation into its adsorption properties for aqueous heavy metal contaminants such as Cr_2_O_7_^2–^. At first, to confirm the anion-exchange procedure and measure the adsorption capacity, 15 mg of PTPA and PTPA–PIP, respectively, were suspended in 8 mL of Cr_2_O_7_^2–^ solutions (500 ppm) at room temperature; the adsorption process was monitored by solution UV–vis spectroscopy based on the typical absorption of Cr_2_O_7_^2–^ at 257 nm. The UV–vis spectra of the Cr_2_O_7_^2–^ solutions before and after adsorption by PTPA and PTPA–PIP for 12 h are shown in Figure 3(a). The Cr_2_O_7_^2^ adsorption peak at 257 nm significantly decreased after exposure to PTPA–PIP, with an accompanying change in the color of the solution from yellow to colorless. As a comparison, despite >3× higher specific area, treatment with PTPA led to virtually no change in the Cr_2_O_7_^2–^ concentration. This striking contrast proves that the driving force for the removal of Cr_2_O_7_^2–^ using PTPA–PIP mainly results from the anion-exchange capability and good hydrophilicity of the polymers.

After adsorption, the Cr-loaded PTPA–PIP material was collected and subjected to XPS investigations to compare PTPA–PIP before and after Cr_2_O_7_^2–^ adsorption. After adsorption, the Cl 2p signal (at 199 eV) disappeared, and two new Cr 2p_1/2_ and Cr 2p_3/2_ peaks were observed (Figure 3(b,c)), proving that the counterions in the skeleton were completely exchanged from Cl^–^ to Cr_2_O_7_^2–^.

The adsorption capacity is calculated by the following formula:
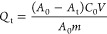
2where *Q*_t_ is the adsorption capacity, *C*_0_ is the initial concentration of the Cr_2_O_7_^2–^ solution, and *A*_0_ and *A*_t_ are the absorbance of the Cr_2_O_7_^2–^ solution at the peak of 257 nm before and after anion exchange, respectively. The removal efficiency of PTPA–PIP for Cr_2_O_7_^2–^ is 86% and the capture capacity is 230 mg g^–1^, which is significantly higher than polyaniline composites such as PANI/CF (18 mg g^–1^)^58^ and polyaniline-coated protonic titanite nanobelts (157 mg g^–1^).^59^ Moreover, this value is also superior to most of the reported cationic MOF materials and possesses one of the highest values for porous materials (see Table S2 for details).

**Figure 4:**
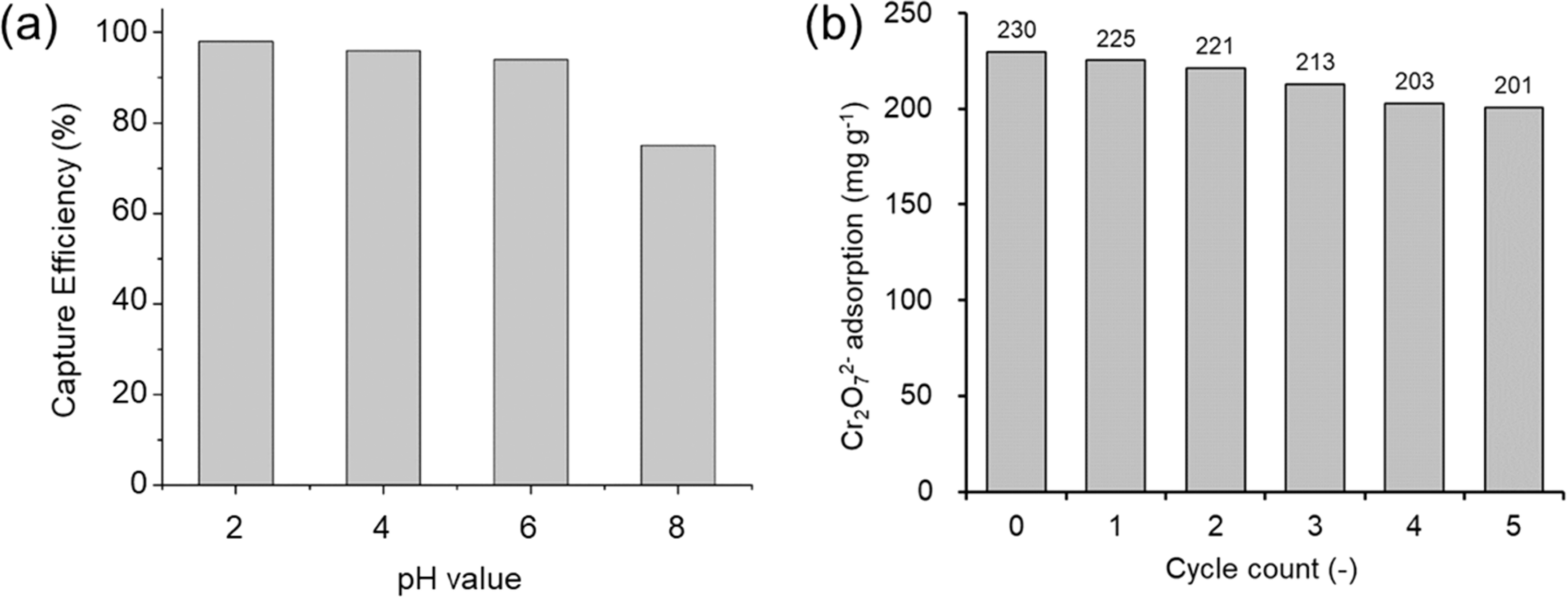
(a) Effect of pH value on the capture efficiency of Cr_2_O_7_^2–^ by PTPA–PIP. (b) Reusability test of PTPA–PIP over repeated cycles.

The effect of pH on Cr_2_O_7_^2–^ adsorption was investigated over the pH range from 2 to 8. As shown in Figure 4(a), the adsorption capacity of PTPA–PIP for Cr_2_O_7_^2–^ decreased slightly and was 98, 96, and 94% at pH values of 2, 4, and 6, respectively. In sharp contrast, however, a significant decrease in the adsorption capacity (76%) was observed at pH 8. This phenomenon could be explained as follows: (a) PTPA–PIP was synthesized by treating PTPA with a strong acid (concentrated hydrochloric acid), thereby protonating the available N atoms, which is not stable in neutral to basic environments. Although there is no direct discussion of the basicity of PTPA in the literature, in the study of Menardo et al.,^60^ the p*K*_a_ values of the structural analogue, polyaniline, were determined to be 2.5 (−NH–, reduced form) and 5.5 (=N–, oxidized form) by titration against NaOH. We hypothesize that a similar phenomenon exists in PTPA. At pH 8, the framework gradually loses its positive charge by deprotonation, which leads to a weakened interaction toward negatively charged Cr(VI) species; (b) it is well known that Cr_2_O_7_^2–^ and CrO_4_^2–^ are interconvertible Cr(VI) forms in aqueous solution. At pH 8, CrO_4_^2–^ is the dominant form.^61^ Comparing the equally (2-) negatively charged Cr_2_O_7_^2–^ and CrO_4_^2–^ species, the fact that CrO_4_^2–^ contains only one Cr atom will lead to less Cr(VI) being captured when equal amounts of adsorption sites are occupied with both ions.

To further explore the practical application of these high-performing POPs, we investigated the reusability of PTPA–PIP. The Cr_2_O_7_^2–^-loaded samples were carefully washed with 3 × 8 mL of 3 M HCl solution and then exposed to fresh Cr_2_O_7_^2–^-containing solutions again (500 ppm, 8 mL). It was found that the polymer could be efficiently reused for at least five cycles, still retaining uptake capacities of >200 mg g^–1^ (Figure 4(b)).

Inspired by the high adsorption capacity, the adsorption kinetics of Cr(VI) by PTPA–PIP, as an important index of the capture efficiency, was further investigated. As shown in Figure S5(a,b), a pseudo-second-order model was found to fit the experimental data better with an *R*^2^ value of 0.99, while a pseudo-first-order model was fitted with a slightly lower *R*^2^ value of 0.98, implying that adsorption of Cr_2_O_7_^2–^ to PTPA–PIP is dominated by both chemical and physical adsorption mechanisms. As shown in Figure S5(b), the amount adsorbed reached 86% of the maximum adsorption capacity after the first 2 min, and after 10 min, no further variation in Cr_2_O_7_^2–^ concentration was observed with extension of contact time, indicating that the anion exchange approaches equilibrium within 10 min. In addition, the initial adsorption rate is very high at 82.9 mg g^–1^ min^–1^, illustrating that PTPA–PIP is capable of ultrafast capture of Cr_2_O_7_^2–^. To our knowledge, the adsorption rate is one of the fastest compared to other POPs and MOFs (see Table S2 for details).

To investigate the effect of the Cr_2_O_7_^2–^ concentration on the capture process, the adsorption isotherm of Cr_2_O_7_^2–^ describing the relationship between the capture capacity (*Q*_e_) and the equilibrium concentration (*C*_e_) was examined (Figure S6). First, a sharp increase of the adsorption capacity at the initial concentration is observed, followed by a concentration-independent process, after which the adsorption reaches saturation. The adsorption isotherm could be fitted well by the Langmuir isotherm model (*R*^2^ > 0.97), indicating a monolayer adsorption on the network surface, with the maximum Cr_2_O_7_^2–^ adsorption reaching up to 230 mg g^–1^.

**Figure 5:**
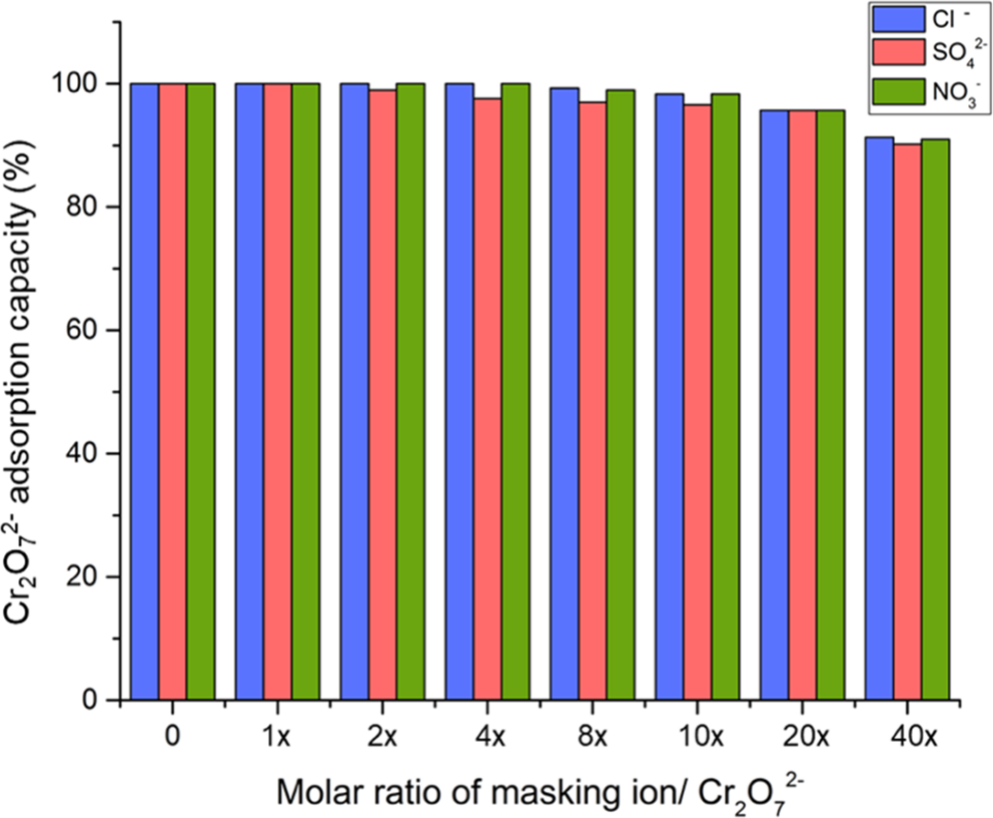
Effect of a single competing ion (Cl ^–^, NO_3_^–^, and SO_4_^2–^) on the capture capacity of PTPA–PIP.

Wastewater typically contains different competing anions (e.g., Cl^–^, SO_4_^2–^, and NO_3_^–^) along with the targeted Cr_2_O_7_^2–^ anions; exploring the selectivity toward Cr_2_O_7_^2–^ is therefore indispensable for real-world application. We therefore performed binary mixture studies for Cr_2_O_7_^2–^ with different competing anions, with the concentration of the competing anions ranging from 1 to 40 times as high as that of Cr_2_O_7_^2–^. As shown in Figure 5, the adsorption capacity for Cr_2_O_7_^2–^ shows almost no change when the concentration of competing anions is 4 times higher than that of Cr_2_O_7_^2–^. Notably, when the concentration of competing anions is 40 times higher than that of Cr_2_O_7_^2–^, only a slight decrease (less than 10%) of the adsorption capacity for Cr_2_O_7_^2–^ was observed. It is worth acknowledging the importance of considering other prevalent ions in wastewater, including phosphate anions. While our current work focused primarily on the dynamics of chromium removal, recognizing the relevance of other species, such as phosphates, underscores the expansive potential of PTPA–PIP in addressing a wider spectrum of pollutants.

The influence of the existence of multiple competing anions was also studied. A Cr_2_O_7_^2–^ solution containing all three types of competing ions was prepared (Cr_2_O_7_^2–^ concentration: 100 ppm; concentration of competing ions: 10 times) and tested against PTPA–PIP. In this challenging case, the adsorption capacity for Cr_2_O_7_^2^ was maintained at a high level of 92%. These results show that PTPA–PIP possesses superior selectivity for Cr_2_O_7_^2^, even when high concentrations of competing anions are present in the solution.

**Scheme 1:**
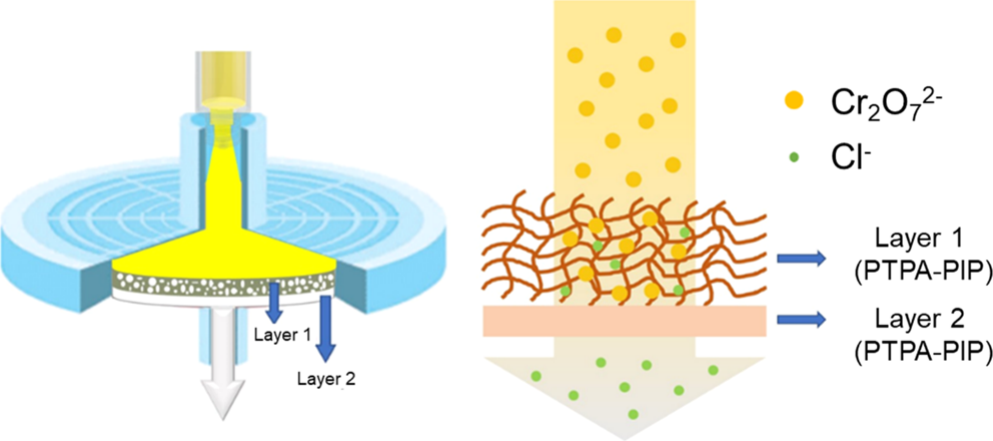
Schematic Representation of the Syringe Filter Structure with Our Active PTPA–PIP Material, and the Anion-Exchange Procedure

Finally, for practical application demonstration, a syringe filter was prepared by loading 10 mg of PTPA–PIP into a 0.45 μm syringe filter (25 mm diameter) and employed for the removal of Cr_2_O_7_^2–^ from water (see Scheme 1). As shown in the demonstration video (Videos S1 and S2), 5 mL of a 100 ppm solution of Cr_2_O_7_^2–^ was loaded into a syringe and injected through the modified filter at an approximate injection rate of 0.35 mL s^–1^. A distinct color change was noticed in the filtrate, as a consequence of Cr_2_O_7_^2–^ being captured by PTPA–PIP, with the UV–vis spectrum of the filtrate revealing the absence of Cr(VI) (Figure S7). As discussed earlier, the filters could be regenerated with 3 M HCl, and a similar capture performance could be observed for at least 5 cycles. It is worth mentioning that the eluted Cr(VI) ions may be collected and further reduced to Cr^3+^ species (e.g., hydrated or nonhydrated Cr_2_O_3_) for efficient detoxification, which is a common practice in industry. These metal oxides are stable under ambient conditions and can be disposed of, depending on the local regulations and laws. Furthermore, as a control experiment, a syringe filter without PTPA–PIP was also tested and no uptake of Cr_2_O_7_^2^ was observed. This result not only shows the durability and performance of PTPA–PIP as an absorbent but also sheds light on a potential new design for small-scale repeat use, which is simple, feasible, and lightweight.

Additionally, an approximate calculation of the raw material expense for our metal-free, reusable materials has been made. Derived from small-scale laboratory production without scale-up or process optimization and using chemicals sourced at standard lab supplier rates (not in bulk), this calculation suggests an approximate price of $1.65 per gram.

## 4 Conclusions

In this study, we successfully introduced a postsynthesis treatment to prepare doped porous polyaniline (PTPA–PIP). This approach provides a facile and cost-effective avenue for the synthesis and use of cationic POPs. This transformation allows tunability of PTPA–PIP’s hydrophilicity and consequent dispersion in water and use in a wide range of environmental applications. Hydrophilic PTPA–PIP exhibits not only rapid uptake, large capture capacity, high removal efficiency, and excellent stability in removing Cr_2_O_7_^2–^ from water but also remarkable selectivity for Cr_2_O_7_^2–^ even in the presence of a large excess of competing anions. We believe that these findings, in addition to the demonstration of a simple and effective syringe filter for personal use, will provide helpful insights and new possibilities for the development of advanced materials for the removal of heavy metal ions and environmental protection, further contributing to safeguarding ecosystems and providing access to safe and clean drinking water.

## Supporting Information Available

Characterization results and comparison of material performances (PDF)
Filtering Cr_2_O_7_^2–^ solution using a syringe filter with PTPA-PIP (AVI)
Filtering Cr^2^O_7_^2–^ solution using a syringe filter without PTPA-PIP (AVI)

## Supplementary Material

ap4c00658_si_001.pdf

ap4c00658_si_002.avi

ap4c00658_si_003.avi
