# Supplementary material for: Polyaniline-Based Cationic Porous Organic Polymers for Fast and Efficient Anion-Exchange-Driven Capture of Cr2O72–
Source: ACS Appl Polym Mater. 2024 May 24;6(11):6416–24. doi: 10.1021/acsapm.4c00658 (PMC11186002; doi:10.1021/acsapm.4c00658)
Supplement: Supplementary file 1 — ap4c00658_si_001.pdf [file ap4c00658_si_001.pdf]

Supporting Information for

Polyaniline-Based Cationic Porous Organic Polymers for Fast and Efficient Anion-Exchange-Driven Capture of $Cr_2O_7^{2-}$

Long Pan,[a,b] *Zilu Liu,*[c] *Marcos Villeda Hernandez,*[a]*, Bob C. Schroeder,*[c] *Yuchen Sun,*[b] *Charl F. J. Faul.* [a,*]

[a]School of Chemistry, University of Bristol, Bristol, England BS8 1TS, U.K.

[b]Institute for Advanced Pharmaceutical Materials, Asymchem Life Sciences (Tianjin) Co., Ltd., No.265 South Avenue, TEDA, Tianjin, 300462, P.R. China

[c]Department of Chemistry, University College London, London WC1H 0AJ, U.K.

*Email for corresponding author: Charl.Faul@bristol.ac.uk

Table of Contents

Figures

Figure S1. SEM images of PTPA-PIP.

Figure S2. Solid-state ^{13}C CPMAS NMR spectra of PTPA and PTPA-PIP.

Figure S3. Zeta potential measurement for PTPA and PTPA-PIP.

Figure S4. N_2 adsorption–desorption isotherms of PTPA and PTPA-PIP measured at 77 K.

Figure S5. Adsorption amount of $Cr_2O_7^{2-}$ with time fitted with pseudo second order model.

Figure S6. Equilibrium adsorption isotherm of $Cr_2O_7^{2-}$ onto PTPA-PIP with Langmuir fits.

Figure S7. UV spectrums of $Cr_2O_7^{2-}$ solutions, before and after removal by syringe filter loaded with PTPA-PIP.

Tables

Table S1. Method of polymerization for preparing porous organic polymers (POPs) reported in literatures.

Table S2. Comparison of $Cr_2O_7^{2-}$ removal of several materials reported in literatures.

Videos

Video S1. Filtering $Cr_2O_7^{2-}$ solution using a syringe filter with PTPA-PIP

Video S2. Filtering $Cr_2O_7^{2-}$ solution using a syringe filter without PTPA-PIP

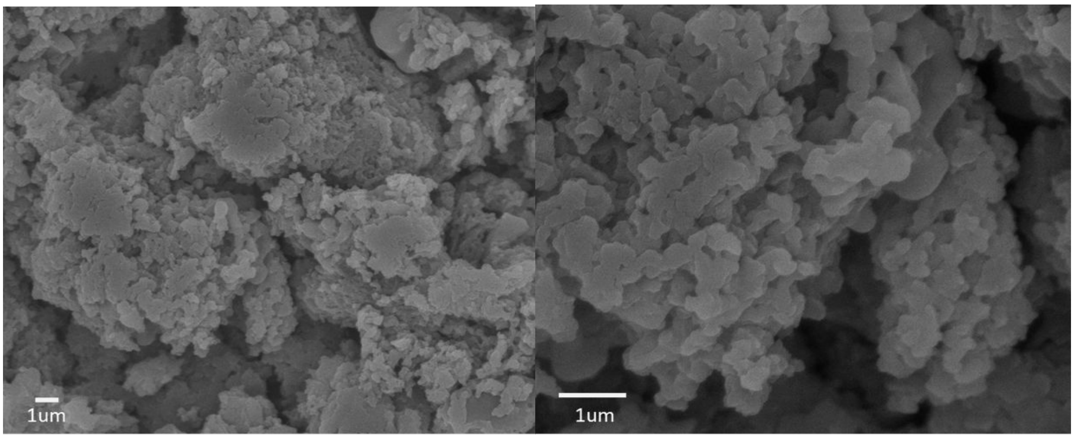

Figure S1. SEM images of PTPA-PIP.

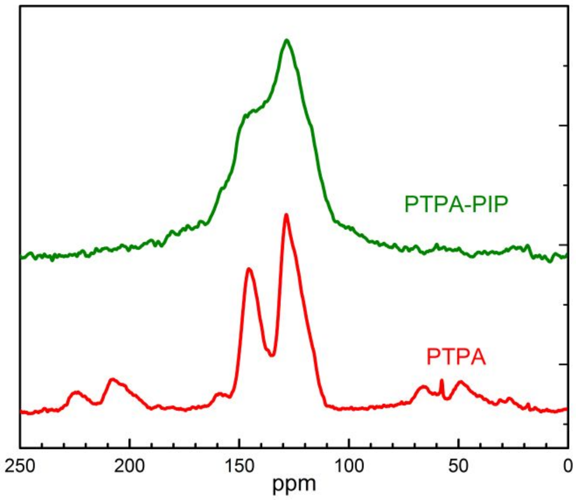

Figure S2. Solid-state ^{13}C CPMAS NMR spectra of PTPA and PTPA-PIP.

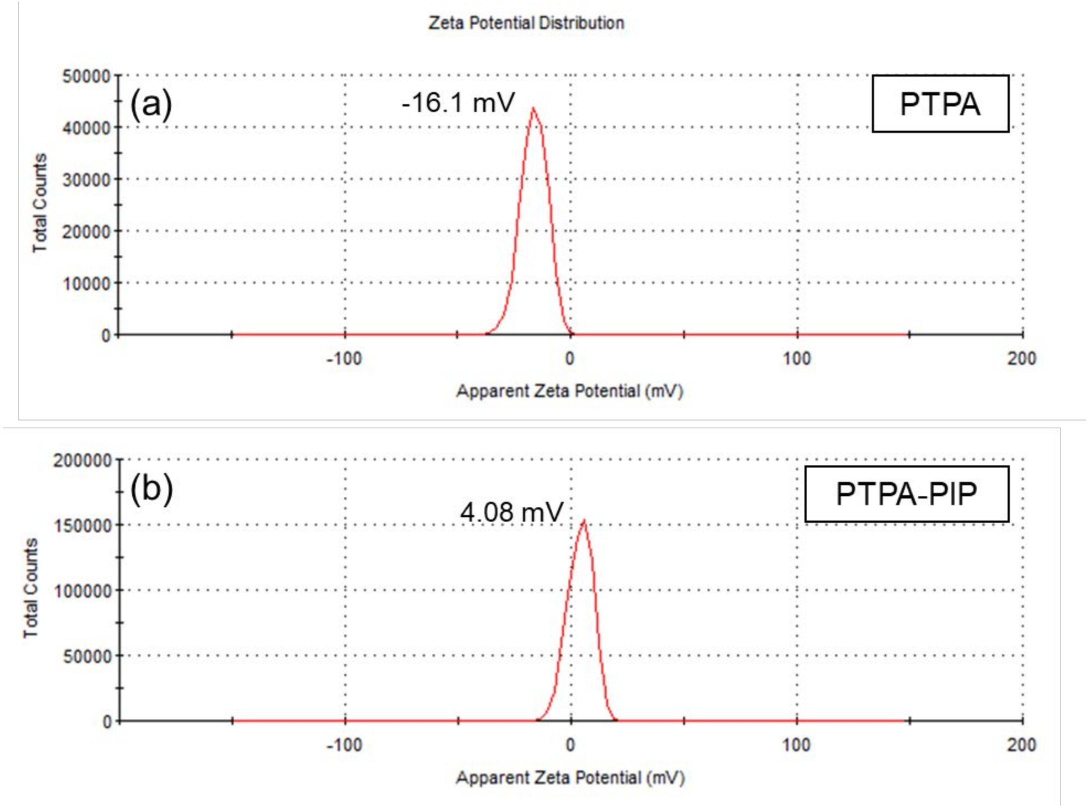

Figure S3. Zeta potential measurement for (a) PTPA and (b) PTPA-PIP.

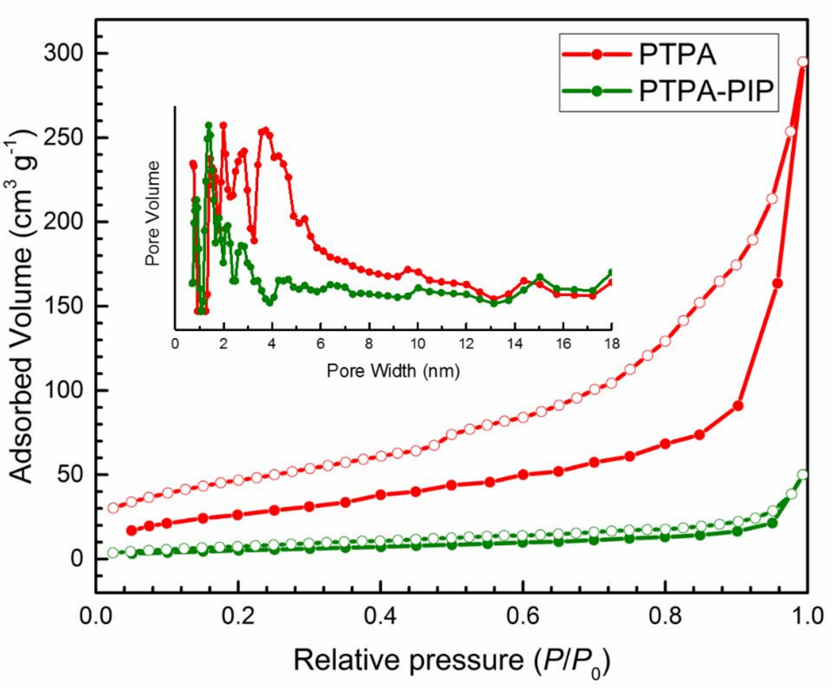

Figure S4. N_2 adsorption–desorption isotherms of PTPA and PTPA-PIP measured at 77 K; the adsorption and desorption branches are marked with solid and open symbols, respectively. Inset: the pore size distribution profiles of PTPA and PTPA-PIP calculated by NLDFT.

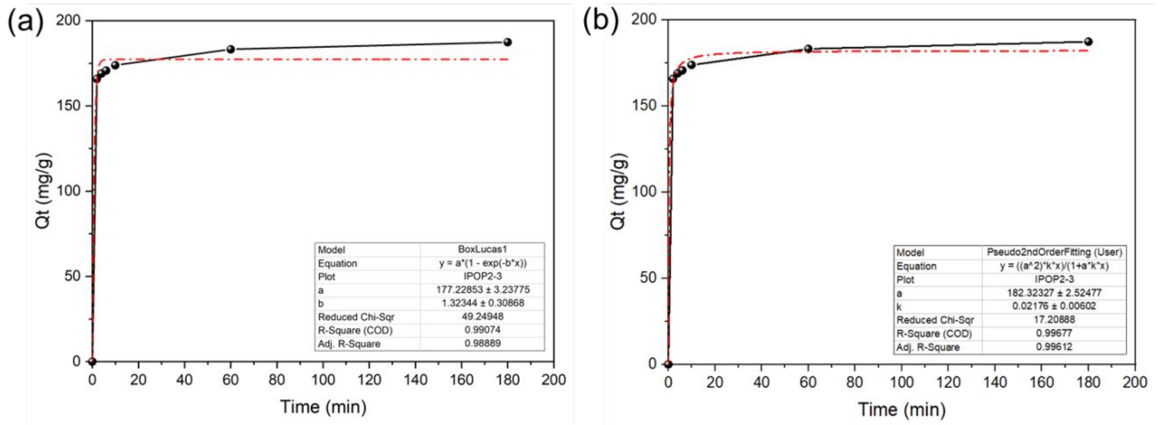

Figure S5. Adsorption amount of $Cr_2O_7^{2-}$ with time fitted with (a) pseudo first-order model and (b) pseudo second-order model.

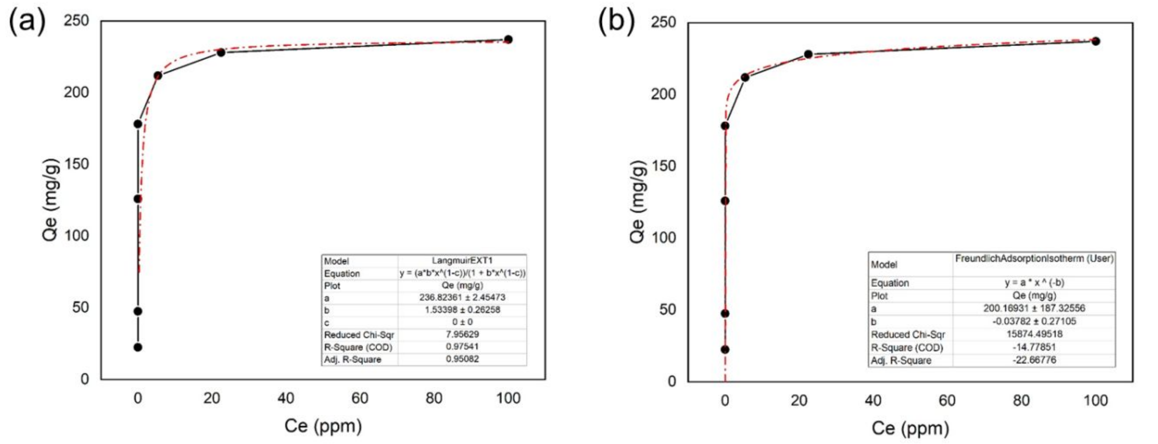

Figure S6. Equilibrium adsorption isotherm of $Cr_2O_7^{2-}$ onto PTPA-PIP with (a) Langmuir and (b) Freundlich fits.

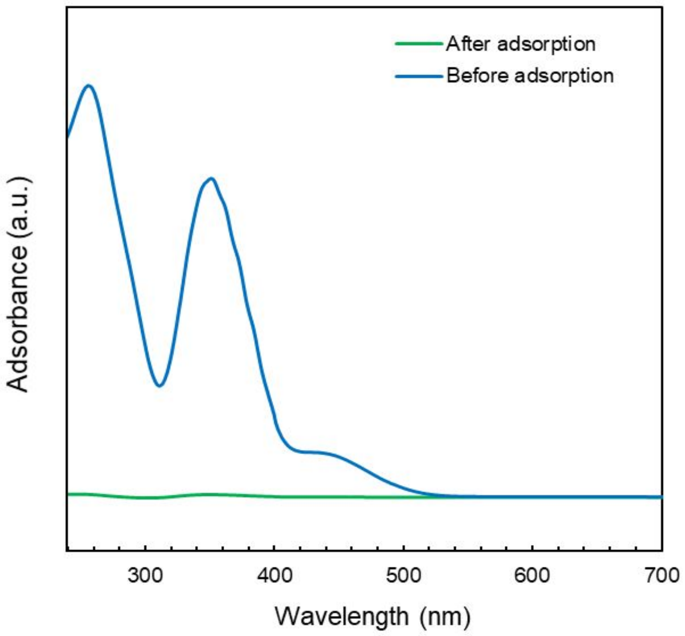

Figure S7. UV spectrums of $Cr_2O_7^{2-}$ solutions, before and after removal by syringe filter loaded with PTPA-PIP.

Table S1. Method of polymerization for preparing porous organic polymers (POPs) reported in literature.

<table>
<tr><td colspan="5">Direct self-polymerization</td></tr>
<tr><td>PIPs</td><td colspan="2">Monomer</td><td>Synthesis method</td><td>Ref</td></tr>
<tr><td>PQP</td><td colspan="2">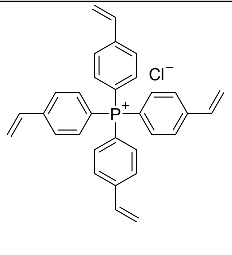</td><td>Free radical polymerization</td><td>[1]</td></tr>
<tr><td>PAF-50</td><td colspan="2">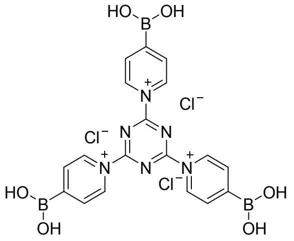</td><td>Condensation</td><td>[2]</td></tr>
<tr><td colspan="5">Copolymerization of ionic and neutral building blocks</td></tr>
<tr><td>PIPs</td><td>Ionic building block</td><td>Neutral monomer</td><td>Synthesis method</td><td>Ref</td></tr>
<tr><td>CPN-1-Br</td><td>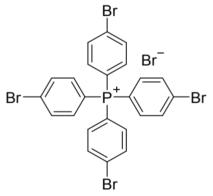</td><td>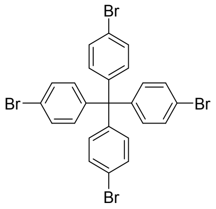</td><td>Yamamoto coupling</td><td>[3]</td></tr>
<tr><td>PC-COF</td><td>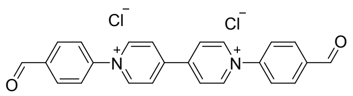</td><td>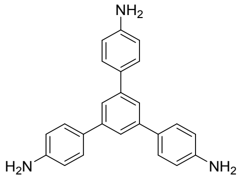</td><td>Schiff-base reaction</td><td>[4]</td></tr>
</table>

Table S2. Comparison of $Cr_2O_7^{2-}$ removal by materials reported in literature.

Adsorbent name	Functional Group	Surface area (m^2 g^{-1})	Q_{max}, $Cr_2O_7^{2-}$ (mg g^{-1})	Initial adsorption rate (mg g^{-1} min^{-1})	Reference s
POPs					
PTPA-PIP	Secondary and tertiary amines	25	230	83	This work
compound-1	N-substituted pyridine	No description	133	50	[5]
POP-Im1	Imidazolium	Negligible	172	80	[6]

H-CMP-A2	Primary, secondary and tertiary amine groups	132	73	40	[7]
QUST-iPOP-1	Imidazolium	274	396	60	[8]
TPP-ThZ CMP	Pyridine	41	209	<10	[9]
CMX-5	Pyridine	No description	493	25	[10]
DA-POP-1	N/A (pseudochemical adsorption)	1041	283	<10	[11]
IISERP-POF11	Phenolic OH, N-substituted pyridine	No description	131	<10	[12]
MOFs					
1-SO_4	N/A	No description	166	<10	[13]
ABT·2ClO_4	N/A	No description	201	Not reported Average adsorption rate within the first hour: 108 mg g^{-1} h^{-1}	[14]
SC–SC	N/A	No description	207	<10	[15]
1-Br	N/A	No description	128	<10	[16]
JLU-MOF60	N/A	345	149	38	[17]
JLU-MOF50	N/A	1101	92	33	[18]
aMOC-1	N/A	25	203	<10	[19]
[Cu_3Cl(L)(H_2O)$_2$]·Cl·4DMA·8H_2O	N/A	No description	70	<10	[20]
Zr-Sti	N/A	No description	43	Not reported	[21]
MOF-808-OH	N/A	2611	164	80	[22]
Other materials					
CS/$CNT_{10:1}$ (nanofiber)	-OH, primary NH_2, g-C_3N_4, TiO_2	No description	239	<10	[23]
OSNCs-3.0wt%PEI (nanocrystal)	Primary, secondary and tertiary amine groups	No description	184	<10	[24]
SBA (coordination polymer)	N/A	No description	297	88	[25]

References:

1. Lei, Y.; Fan, M.; Lan, G.; Li, G., Copper supported on N-heterocyclic carbene-functionalized porous organic polymer for efficient oxidative carbonylation of methanol. *Applied Organometallic Chemistry* **2020,** *34* (9), e5794.

2. Yuan, Y.; Sun, F.; Li, L.; Cui, P.; Zhu, G., Porous aromatic frameworks with anion-templated pore apertures serving as polymeric sieves. *Nature communications* **2014,** *5* (1), 4260.

3. Fischer, S.; Schimanowitz, A.; Dawson, R.; Senkovska, I.; Kaskel, S.; Thomas, A., Cationic microporous polymer networks by polymerisation of weakly coordinating cations with CO_2-storage ability. *Journal of Materials Chemistry A* **2014,** *2* (30), 11825-11829.

4. Yu, S.-B.; Lyu, H.; Tian, J.; Wang, H.; Zhang, D.-W.; Liu, Y.; Li, Z.-T., A polycationic covalent organic framework: a robust adsorbent for anionic dye pollutants. *Polymer Chemistry* **2016,** *7* (20), 3392-3397.

5. Samanta, P.; Chandra, P.; Dutta, S.; Desai, A. V.; Ghosh, S. K., Chemically stable ionic viologen-organic network: an efficient scavenger of toxic oxo-anions from water. *Chemical Science* **2018,** *9* (40), 7874-7881.

6. Su, Y.; Wang, Y.; Li, X.; Li, X.; Wang, R., Imidazolium-based porous organic polymers: anion exchange-driven capture and luminescent probe of $Cr_2O_7^{2-}$. *ACS Applied Materials & Interfaces* **2016,** *8* (29), 18904-18911.

7. Ko, J. H.; Lee, S. M.; Kim, H. J.; Ko, Y.-J.; Son, S. U., Skeleton carbonylation of conjugated microporous polymers by osmium catalysis for amine-rich functionalization. *ACS Macro Letters* **2018,** *7* (11), 1353-1358.

8. Jiao, S.; Deng, L.; Zhang, X.; Zhang, Y.; Liu, K.; Li, S.; Wang, L.; Ma, D., Evaluation of an ionic porous organic polymer for water remediation. *ACS Applied Materials & Interfaces* **2021,** *13* (33), 39404-39413.

9. Kotp, M. G.; Torad, N. L.; Nara, H.; Chaikittisilp, W.; You, J.; Yamauchi, Y.; EL-Mahdy, A. F.; Kuo, S.-W., Tunable thiophene-based conjugated microporous polymers for the disposal of toxic hexavalent chromium. *Journal of Materials Chemistry A* **2023,** *11* (27), 15022-15032.

10. Elgamal, A. M.; Abd El-Ghany, N. A.; Saad, G. R., Highly reactive adsorbent based on carboxymethyl xanthan gum-g-poly (4-vinylpyridine) copolymer for the potential removal of Acid Orange 10 dye and Cr (VI) ions for water treatment. *Journal of Applied Polymer Science* **2022,** *139* (47), e53179.

11. Cao, Z.; Wang, M.; Gao, H.; Li, L.; Ren, S., Porous Organic Polymers via Diels–Alder Reaction for the Removal of Cr (VI) from Aqueous Solutions. *ACS Macro Letters* **2022,** *11* (4), 447-451.

12. Chakraborty, D.; Nandi, S.; Kushwaha, R.; Kaleeswaran, D.; Vaidhyanathan, R., Viologen functionalized CC bonded cationic polymers for oxo-anion pollutant removal from aqueous medium. *Materials Research Bulletin* **2022,** *146*, 111614.

13. Desai, A. V.; Manna, B.; Karmakar, A.; Sahu, A.; Ghosh, S. K., A water-stable cationic metal–organic framework as a dual adsorbent of oxoanion pollutants. *Angewandte Chemie* **2016,** *128* (27), 7942-7946.

14. Li, X.; Xu, H.; Kong, F.; Wang, R., A cationic metal–organic framework consisting of nanoscale cages: capture, separation, and luminescent probing of $Cr_2O_7^{2-}$ through a single-crystal to single-crystal process. *Angewandte Chemie International Edition* **2013,** *52* (51), 13769-13773.

15. Li, C.-P.; Zhou, H.; Wang, S.; Chen, J.; Wang, Z.-L.; Du, M., Highly efficient $Cr_2O_7^{2-}$ removal of a 3D metal-organic framework fabricated by tandem single-crystal to single-crystal transformations from a 1D coordination array. *Chemical Communications* **2017,** *53* (66), 9206-9209.

16. Lv, X.-X.; Shi, L.-L.; Li, K.; Li, B.-L.; Li, H.-Y., An unusual porous cationic metal–organic framework based on a tetranuclear hydroxyl-copper (II) cluster for fast and highly efficient dichromate trapping through a single-crystal to single-crystal process. *Chemical communications* **2017,** *53* (11), 1860-1863.

17. Liu, J.; Ye, Y.; Sun, X.; Liu, B.; Li, G.; Liang, Z.; Liu, Y., A multifunctional Zr (iv)-based metal–organic framework for highly efficient elimination of Cr (vi) from the aqueous phase. *Journal of materials chemistry A* **2019,** *7* (28), 16833-16841.

18. Sun, X.; Yao, S.; Yu, C.; Li, G.; Liu, C.; Huo, Q.; Liu, Y., An ultrastable Zr-MOF for fast capture and highly luminescence detection of $Cr_2O_7^{2-}$ simultaneously in an aqueous phase. *Journal of Materials Chemistry A* **2018,** *6* (15), 6363-6369.

19. Jin, X.; Wang, G.-Q.; Ma, D.; Deng, S.-Q.; Cai, S.-L.; Fan, J.; Zhang, W.-G.; Zheng, S.-R., Cationic amorphous metal–organic cage-based materials for the removal of oxo-anions from water. *ACS Applied Nano Materials* **2019,** *2* (9), 5824-5832.

20. Li, Y.; Yang, J.; Ma, J.-F., A copper (ii)-based porous metal–organic framework for the efficient and rapid capture of toxic oxo-anion pollutants from water. *Dalton Transactions* **2021,** *50* (11), 3832-3840.

21. Yin, R.; Bu, Y.; Zhu, H.; Su, P.; Ye, E.; Li, Z.; Loh, X. J.; Yuan, C.; Wang, S., Simultaneous detection and removal of 2, 4, 6-trinitrophenyl phenol and dichromate by metal-organic framework. *Spectrochimica Acta Part A: Molecular and Biomolecular Spectroscopy* **2023,** *297*, 122735.

22. Nguyen, K. D.; Vo, N. T.; Le, K. T.; Ho, K. V.; Phan, N. T.; Ho, P. H.; Le, H. V., Defect-engineered metal–organic frameworks (MOF-808) towards the improved adsorptive removal of organic dyes and chromium (vi) species from water. *New Journal of Chemistry* **2023,** *47* (13), 6433-6447.

23. Li, Q.-H.; Dong, M.; Li, R.; Cui, Y.-Q.; Xie, G.-X.; Wang, X.-X.; Long, Y.-Z., Enhancement of Cr (VI) removal efficiency via adsorption/photocatalysis synergy using electrospun chitosan/g-C_3N_4/TiO_2 nanofibers. *Carbohydrate Polymers* **2021,** *253*, 117200.

24. Chen, Q.; You, N.; Zhao, Y.; Liang, C.; Liu, Z.; Zhao, W., Polyethyleneimine Grafted H2O2-Oxidized Starch Nanocrystals as a Biomaterial for Adsorptive Removal of Cr (VI). *Starch-Stärke* **2022,** *74* (9-10), 2200129.
25. Conour, C. S.; Droege, D. G.; Ehlke, B.; Johnstone, T. C.; Oliver, S. R., Selective Chromium (VI) Trapping by an Acetate-Releasing Coordination Polymer. *Inorganic Chemistry* **2022,** *61* (51), 20824-20833.
